# On the Effect of Sphere-Overlap on Super Coarse-Grained Models of Protein Assemblies

**DOI:** 10.1007/s13361-018-1974-2

**Published:** 2018-05-07

**Authors:** Matteo T. Degiacomi

**Affiliations:** Department of Chemistry, Durham University, South Road, Durham, DH1 3LE UK

**Keywords:** Molecular modeling, Protein assembly, Native mass spectrometry, Ion mobility, super coarse-grain

## Abstract

Ion mobility mass spectrometry (IM/MS) can provide structural information on intact protein complexes. Such data, including connectivity and collision cross sections (CCS) of assemblies’ subunits, can in turn be used as a guide to produce representative super coarse-grained models. These models are constituted by ensembles of overlapping spheres, each representing a protein subunit. A model is considered plausible if the CCS and sphere-overlap levels of its subunits fall within predetermined confidence intervals. While the first is determined by experimental error, the latter is based on a statistical analysis on a range of protein dimers. Here, we first propose a new expression to describe the overlap between two spheres. Then we analyze the effect of specific overlap cutoff choices on the precision and accuracy of super coarse-grained models. Finally, we propose a method to determine overlap cutoff levels on a per-case scenario, based on collected CCS data, and show that it can be applied to the characterization of the assembly topology of symmetrical homo-multimers.

**Figure:**
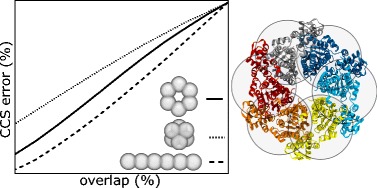
Graphical Abstract

Graphical Abstract

## Introduction

Most proteins assemble into complexes to achieve a specific biological function [1]. Atomic-level information about these complexes can provide precious insights into their mode of action. However, obtaining such high-resolution information is often technically challenging. In this context, integrative modeling approaches can be used to combine low-resolution experimental data on the complex with high-resolution structural information on its subunits, to build models rationalizing all observables [2].

Native ion mobility mass spectrometry (IM/MS) reports on the connectivity between protein subunits and allows deriving the collision cross section (CCS) of these, as well as their sub-complexes [3]. In recent years, efforts have been dedicated to exploit this data within integrative modeling protocols [4, 5]. Unfortunately, sometimes no atomic model of all the subunits of a complex is available. In this case, super coarse-grained models may be adopted, whereby every molecular subunit is represented by one (or a few more) large sphere [6, 7].

The orientation-averaged projected area of an object can be taken as an approximation of its CCS [8]. This approximation includes a hard-sphere contribution given by the radius of the buffer gas used as probe, while ignoring long-range interactions and multiple collisions with it. In the case of folded proteins, it has been shown that, upon scaling, this yields values in good agreement (3% error) with experimental CCS data [9, 10]. When the object under study is convex, its average projected area is equal to a quarter its surface [11]. As such, the radius *r* of a sphere having a CCS equal to the protein it represents, when probed in a drift cell filled with an inert gas having radius *r*_gas_, can be calculated analytically:

1 $$ r(CCS)=\sqrt{\frac{CCS}{\pi }}-{r}_{\mathrm{gas}} $$

**Figure 1:**
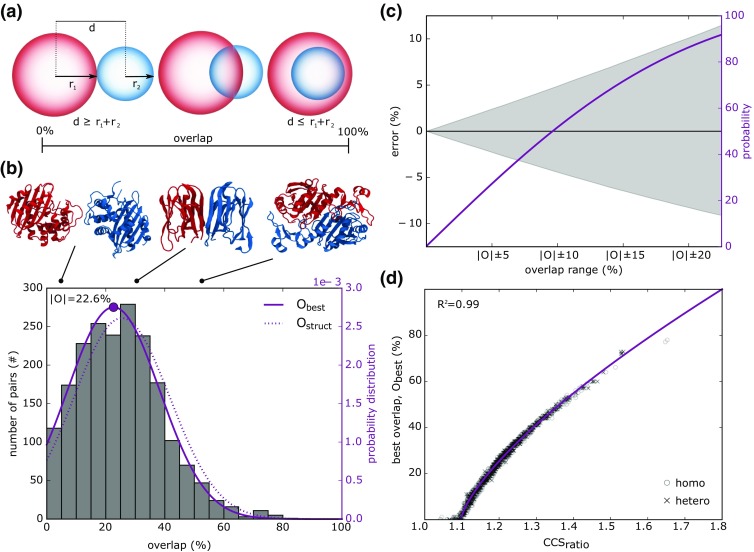
Relationship between sphere-overlap and their CCS (**a**). The overlap between two spheres ranges from 0 (spheres touching or not in contact) to 100% (the smaller sphere is completely embedded in the larger one). (**b**) The histogram shows the distribution of optimal overlap *O*_best_ in our benchmark set of 1988 protein pairs. Data can be fitted with a Gaussian curve (solid line) centered at an overlap level |*O*| equal to 22.6%. As comparison, the Gaussian fitting the distribution of *O*_struct_ is also shown (dotted line). Three proteins, featuring three different overlap levels are shown: lactamase (PDB: 1M6K, O_best_ = 2%), BanLec (PDB: 5EXG, O_best_ = 29%), and the peroxidase HORF6 (PDB: 1PRX, *O*_best_ = 50%). (**c**) The gray area shows the error in CCS value connected to the choice of an overlap interval of a specific size (e.g., |*O*| ± 15 indicates an overlap interval from 7.6 to 37.6%). Each interval choice is connected to a certain likelihood of including the specific *O*_best_ value for the complex under study, shown with a palatinate colored line. (**d**) Relationship between ratio of CCS of individual components and that of complex, against spheres overlap. Given measured CCS of individual components and complex, the ideal overlap between spheres representing protein subunits can be predicted (solid line). The relationship holds independently from the relative radius of the spheres representing the binding partners: both homo- and hetero-multimers are equally distributed along the same trend

The simplest scenario is that of modeling a protein dimer as two spheres using as a guide the CCS of the subunits and that of the resulting complex. Having defined the radius of the two representative spheres as per Eq. 1, the objective is to identify how much these should overlap (or co-penetrate) so that the CCS of the resulting complex has a minimal discrepancy from the experimental value. The overlap has been typically defined as the spheres’ center-to-center distance [6, 7]. However, two spheres would be effectively fully overlapping when the smallest is fully embedded in the largest (Fig. 1a). In this extreme case, the CCS of the complex will be equivalent to that of the largest sphere. Not representing this feature in the definition of sphere-overlap means that the same complex’s CCS will be associated to a range of overlap levels, the size of which will be proportional to the difference in radius between the two interacting spheres. This complicates the definition of an overlap cutoff criterion applicable to any pair of interacting spheres. Given a center-to-center distance *d* of two spheres with radii *r*_1_ and *r*_2_, we suggest the following as a more suitable metric to define their overlap *O*:

2 $$ 0(d)=\left\{\begin{array}{cc}1& if\;d<{r}_1-{r}_2\\ {}0& if\;d>{r}_1+{r}_2\\ {}\frac{r_1+{r}_2-d}{2{r}_2}& \mathrm{otherwise}\end{array},\kern1em \mathrm{with}\;{r}_1\ge {r}_2\right. $$

It should be noted that, in the absence of substantial conformational changes upon binding, it will always be possible to find an overlapping arrangement of two spheres so that their combined CCS matches that of the complex they form.

To assess the relationship between spheres’ overlap and their associated CCS, we selected an ensemble of 1988 protein couples from the PiQSi database [12]. Of these, 241 were crystallized as dimers, whereas the rest were proteins being in contact within 526 crystal structures of larger assemblies. Using IMPACT [9], software numerically estimating the CCS of molecular structures using the projection approximation method, we calculated the CCS of each dimer, as well as that of their constituent subunits. Then, for each pair, we placed a sphere having radius as per Eq. 1 (with *r*_gas_ = 1 Å, representing helium) on the center of mass of each protein subunit and calculated their resulting overlap, hereon called *O*_struct_. Such test has been already performed previously, on smaller datasets, to identify an overlap interval representative of most protein couples [6]. This led to proposing a confidence interval between 15 and 45% for sphere-overlap, usable to guide super coarse-grained integrative modeling protocols exploiting CCS data. Analyzing the average value of *O*_struct_ may however not be perfectly suited to this context. Indeed, integrative modeling protocols typically exploit an optimization engine to find an arrangement of protein subunits minimizing a scoring function usually including terms for the physics of molecular interactions (e.g., van der Waals, electrostatics), and assessments of models’ match against available experimental data. As such, optimizers will be naturally guided to the overlap level *O*_best_ associated to an arrangement of spheres having the smallest deviation from the target dimer CCS. Therefore, for each protein pair, we also tested a range of overlap levels (from 0 to 100%, with steps of 1%), assessing their error with respect of the known dimer CCS, and identifying the optimal overlap *O*_best_ for each of them. For this test, the CCS of each sphere dimer was calculated with IMPACT. The collected *O*_struct_ and *O*_best_ values were both Gaussian distributed and centered at 25.4 ± 16.2 and 22.6 ± 15.6%, respectively (Fig. 1b). Analyzing solely protein pairs generated for dimers, and pairs extracted from larger complexes, yielded similar results.

Any overlap confidence interval used to determine whether a sphere arrangement is suitable will be associated to a CCS error: the larger the interval, the broader the range of accepted CCS values. On the other hand, the wider this interval, the higher the likelihood of including within it the most suitable overlap level. For instance, defining the acceptable overlap interval as being within one standard deviation of *O*_best_ mean value, i.e., anything between 7.0 and 38.2%, is associated to a CCS error of ± 7.4%, and a likelihood of 73.7% of including *O*_best_ in this interval (Fig. 1c). Taken in the context of a modeling framework, this observation indicates there is a non-negligible likelihood for a constraint based on CCS and one based on the statistical distribution of overlaps to be inconsistent. It is therefore not advisable to use such an overlap restraint where CCS data is available.

Marklund has noted that the CCS of a complex can be derived from the CCS of the individual binding partners and their associated orientation-averaged occluded area [13]. Taken in the context of intersecting spheres, since the occluded area depends on sphere-overlap, sphere-overlap and CCS values are connected. Therefore, a suitable overlap confidence interval should be predictable on the basis of given CCS measurements. We observed that the ideal overlap percentage of two spheres is correlated to the ratio of the sum of subunits’ CCS and the complex CCS. Let two molecules, *M1* and *M2*, and *CCS*_*M*1+*M*2_ their CCS when in a complex. We define *CCS*_ratio_ as:

4 $$ {CCS}_{\mathrm{ratio}}=\frac{CCS_{M1}+{CCS}_{M2}}{CCS_{M1+M2}} $$

The relationship between *CCS*_ratio_ and best overlap *O*_best_ can be fitted with the following non-linear model (Fig. 1d):

5 $$ {O}_{\mathrm{best}}=128.67\times {\left({CCS}_{\mathrm{ratio}}-1.1\right)}^{0.71} $$

Here, *CCS*_ratio_ is always greater than 1.09, i.e., the (numerically estimated) minimal possible value associated to spheres being just in contact. We note that this relationship is expected to hold only when treating the overlap of two convex objects. *CCS*_*M1*_, *CCS*_*M2*_, and *CCS*_*M1+M2*_ will all be subjected to a specific experimental error. Using error propagation, the error associated with *CCS*_ratio_ is:

6 $$ err\left({CCS}_{\mathrm{ratio}}\right)={CCS}_{\mathrm{ratio}}\sqrt{{\left(\frac{\sqrt{err{\left({CCS}_{M2}\right)}^2+ err{\left({CCS}_{M2}\right)}^2}}{CCS_{M1}+{CCS}_{M2}}\right)}^2+{\left(\frac{err\left({CCS}_{M1+M2}\right)}{CCS_{M1+M2}}\right)}^2} $$

We calculated *CCS*_ratio_ and *err*(*CCS*_ratio_) for each protein pair in our benchmark dataset, supposing a generous experimental error of 3% on each CCS measure (larger than the typical experimental error [9, 14]). These values allowed us to define, for each protein pair, a custom overlap confidence interval, i.e., an overlap region consistent with data derived by ion mobility spectrometry. On average, the obtained intervals had a size (distance from minimum to maximum acceptable overlap) of 13.1%, i.e., less than half than what is typically considered when adopting the same, statistically determined, interval for all protein dimers. Furthermore, for all pairs, the predicted intervals included their specific *O*_best_ value. Within these intervals, CCS measurements had an average standard deviation of 3.5%. In summary, our data-driven method to define overlap restraints, hereafter called “adaptive cutoff,” is both more precise and accurate than the traditionally used constant cutoff (i.e., same for each case) based upon a statistical analysis of an ensemble of protein pairs.

**Figure 2:**
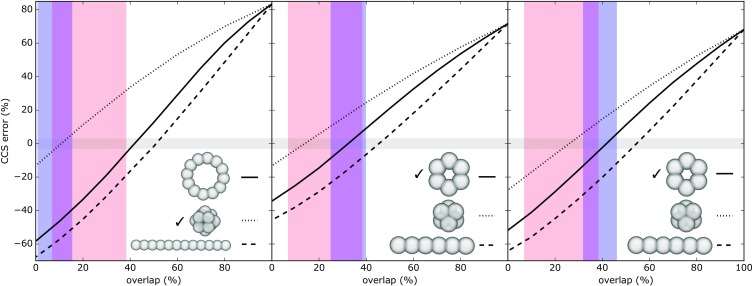
Testing the predictive power of overlap confidence intervals. For three different homo-multimers (from left to right: PDBs 4I88, 1D2N, and 1G41), we produced a range of super coarse-grained models according to different candidate topologies. We then assessed whether the correct topology could be identified (indicated with a tick mark in each case), by filtering the models according to both their CCS matching with the known value (3% error, gray region), and the amount of overlap between their subunits. Red vertical bands indicate overlap confidence intervals defined by the constant cutoff method, blue bands by our adaptive cutoff one, and purple bands regions where both methods agree. To be considered acceptable, a topology must have its trend line within the region at the interception between the gray and red (or blue) areas. The constant cutoff method produced both false positives and false negatives, whereas our adaptive cutoff method always identified the correct topology

We next tested the performance of these two alternative overlap distance restraints for the determination of a macromolecular assembly-specific topology. For this test, we selected three simple cases from the PiQSi database: two forming homo-hexameric circles, and one forming a homo-dodecameric octahedron (i.e., assemblies where all protein-protein interfaces are identical). For each of those, we assessed whether the correct assembly topology could be identified from a range of candidate symmetries (Fig. 2). For each candidate topology, we generated a range of assemblies with varying overlap level. An assembly model would be considered valid (i.e., a specific topology would explain the data) if it had a CCS error < 3%, and the overlap of its constituting spheres was within a designated confidence interval. When using the constant cutoff method, the octahedral topology could be correctly identified, for one of the two hexamers a false positive was obtained (both tetrahedron and circle were considered plausible) and for the other a false negative was produced (tetrahedron instead of circle). With our adaptive cutoff method, all three cases were instead unambiguously assigned to the correct topology. Using a CCS cutoff smaller than 3% would have increased the errors in the case of the statistics-based overlap, but not in the case of our adaptive method.

In conclusion, we suggest Eq. 2 to be a more suitable metric to define the overlap between two spheres representing super coarse-grained models of proteins. When information about the CCS of both spheres and their complex is available, our adaptive cutoff method should be used to define a suitable confidence interval for the overlap between two spheres, with the overlap defined as per Eq. 1. We note that, in case binding leads to conformational changes altering the CCS of the individual binding partners, the adaptive cutoff will impose a tighter or looser sphere-overlap level. When no information about the CCS of both spheres and their complex is available, the confidence interval should be instead defined on the basis of the constant cutoff criterion we determined by analyzing a large protein pair dataset. The mean overlap value we determined here is Gaussian distributed at 22.6 ± 15.6%. We have however observed that the identification of a protein assembly topology applying such a cutoff on spheres overlap is prone to both false negatives and false positives. Still, we should stress that our tests were simple cases based on symmetrical homo-multimers. It cannot be excluded that better performances may be observed when modeling larger hetero-multimers with no symmetry. Our data-driven adaptive cutoff led to accurate topology prediction in all test cases. This method suffers of two limitations: (1) it currently only applies to symmetrical homo-multimers and, (2) besides the CCS of a single building block and the whole complex, it also requires the CCS of both a monomer and a dimer. Nevertheless, we believe that our observations indicate that exploiting experiment-based overlap restraints for the characterization of protein assembly topologies is a promising route for substantially increasing super coarse-grained models’ accuracy.
